# Usefulness of Midline Catheters versus Peripheral Venous Catheters in an Inpatient Unit: A Pilot Randomized Clinical Trial

**DOI:** 10.3390/nursrep12040079

**Published:** 2022-10-31

**Authors:** Marcela Villalba-Nicolau, Elena Chover-Sierra, Carlos Saus-Ortega, Maria Luisa Ballestar-Tarín, Pilar Chover-Sierra, Antonio Martínez-Sabater

**Affiliations:** 1 Consultorio de Motilleja, Centro de Salud de Madrigueras, Gerencia de Arencion integrada de Albacete, 02230 Albacete, Spain; 2 Nursing Department, Facultat d’Infermeria i Podologia, Universitat de València, 46010 Valencia, Spain; 3 Internal Medicine, Consorcio Hospital General Universitario de Valencia, 46014 Valencia, Spain; 4 Nursing Care and Education Research Group (GRIECE), GIUV2019-456, Nursing Department, Universitat de Valencia, 46010 Valencia, Spain; 5 Nursing School La Fe, Adscript Center of Universidad de Valencia, 46026 Valencia, Spain; 6 Grupo Investigación en Cuidados (INCLIVA), Hospital Clínico Universitario de Valencia, 46010 Valencia, Spain

**Keywords:** midline catheter, peripheral intravenous catheter (PIVC), catheter-related infections, phlebitis, patient safety

## Abstract

Canalization of vascular accesses is one of the most used techniques in hospitalization units. When talking about peripherally inserted catheters, we can differentiate between peripheral intravenous catheters (PIVC), midline catheters, and long peripheral catheters (LPC). Midline catheters are rarely used despite being recommended for intravenous therapies lasting more than six days. This research is a pilot study of a longitudinal clinical trial. It aims to compare the complications associated with intravenous therapy between the control group (CG) with a PIVC and the experimental group (EG) with a midline in an Internal Medicine Unit of a Spanish hospital for three months. In this study, 44 subjects participated, 25 in the CG and 19 in the EG. The duration of cannulation was longer in the experimental group (8.13 days vs. 3.22, *p* < 0.001), and the appearance of phlebitis was more significant in the control group (19 patients in CG and 25 patients in EG). Midlines have presented a longer duration of cannulation and fewer complications than the PIVC. This protocol was registered with ClinicalTrials.gov (NCT05512117).

## 1. Introduction

Vascular access is the placement of a plastic cannula into a vein for infusion of fluids, medication, blood product, or nutrition and it is one of the most used techniques at all levels of care [1], peripheral intravenous cannulation (PIVC) being the most frequent one [2]. The goals of infusion therapy are to preserve vascular health [3] and to administer needed treatment [4] safely. The selection of the most appropriate vascular access device (VAD) is necessary to avoid potentially severe complications of infection and/or thrombosis, so indications and recommendations must be considered [4]. The insertion and care of catheters are nurses’ responsibilities. They become a role model for both students in training and other professionals in their daily practice [5]. For this reason, implementing evidence-based practices on a day-to-day basis improves satisfaction and service outcome indicators, including economic repercussions associated with cost reduction [6,7,8,9,10]. It also impacts patients’ quality of life [11,12].

Peripheral venous catheters can be subdivided based on their length: PIVC (short), from 3 to 6 cm; long peripheral catheters (LPC) with a length of 6 to 15 cm; and midline catheters which are from 15 cm to 25 cm. LPC and midline are indicated for more than 6 days of intravenous treatments [13,14], being almost necessary for situations such as hematopoietic transplants and chemotherapy treatments [15].

Peripherally inserted central catheters (PICC) and midline are frequently used for venous access in the medium and long term [16]. Its use is increasing day to day [17], so it is necessary to implement studies to assess this use of midline catheters to reduce the need to insert central catheters or repeated peripheral catheters [18]. In different short-term monitoring studies, midline use was associated with a lower risk of infection and bloodstream occlusion versus PICC use. The use of different long-term systems [16,19] may have advantages in the quality of life in specific cases, such as children [20,21], people with difficulties to access peripheral vasculature [9,22], or chronic situations [23] by reducing the need for punctures and their associated complications as well [4]. The impact on quality of life due to vascular access complications has been measured in different studies [23,24,25,26].

The use of catheters is not exempt from complications that are associated with variables such as insertion time, the use of irritating drugs, etc. [27,28]. Minor complications include phlebitis, infiltrations, and tearing, with major complications as infections related to vascular catheters standing out for their relevance in morbidity and mortality [29].

Midlines are versatile venous access devices that appear to be effective and safe for short-term vascular access in patients requiring peripherally compatible infusates. Midlines could be characterized by their long dwell time and high rate of first-attempt placement. Their rate of major complications is low, although minor complications necessitating device removal are common. As mentioned above, its use in specific conditions could reduce patient costs and risks [7,8,10]. The patient’s clinical status, the ongoing need for laboratory tests, and the intravenous therapy used should guide the selection of size, type, and placement of intravenous devices [8,10].

The process of an adequate selection of the catheter must be accompanied by educational interventions aimed at the nursing staff. Some strategies have been implemented, including educational activities and simple measures that have shown great efficacy in preventing catheter-related complications when applied to high-risk patients [30,31]. Along with these educational actions, some others must be included, such as training professionals in inserting and maintaining catheters and monitoring and recording possible complications [27]. Different studies influence the promotion of these good practices to improve device management [32], finding the discrepancies between the daily maintenance practices and hospital protocols and policies that can justify high rates of complications [5,33].

Different studies indicate that patients of medical specialties would benefit most from the implantation of medium-duration catheters [8]. The standard inpatient of our department is identified with the following characteristics: average stay of fourteen days, multiple intravenous treatments, and difficulties for cannulation due to capillary fragility. This patient is an ideal candidate for the insertion of a midline catheter, intending to avoid repeated punctures and complications.

Given the importance of knowing the situation of the Internal Medicine Unit of the Consorcio Hospital General Universitario de Valencia, the objective of this study was to analyze complications and length of insertion of midline catheters and compare these with complications and length of insertion of conventional peripheral catheters.

With this objective, an experimental study was designed with the hypothesis that the rate of complications of midline catheters would be similar to or lower than that of peripheral catheters. At the same time, the number of punctures would be less as the canalization’s duration of the catheter was increased.

## 2. Methods

### 2.1. Trial Design

A randomized, parallel, and longitudinal trial with two groups. Subjects in the experimental group were inserted with a midline catheter when necessary, while subjects in the control group received a conventional peripheral catheter.

Data related to the characteristics (i) of the catheters (the type of catheter: PIVC or midline, the duration of insertion, the appearance of complications, the reason for removal) and (ii) of the subjects (age, gender, reason for admission, comorbidity index) were collected through an ad hoc questionnaire.

### 2.2. Participants

The patients included were those hospitalized in the Internal Medicine Service of the Consorcio Hospital General Universitario de Valencia who needed intravenous therapy and who had signed the Informed Consent for placement of midline at the time of the change of the PIVC placed in the emergency unit.

Within the first 24 h of hospitalization, each patient hospitalized in the unit and their family received a document that briefly explained what the study consisted of, the title, the risks and benefits, what participating in the study implied, and its confidentiality. In addition, the unit nurses could solve the study’s doubts or transfer them the next day to the researchers. Along with this document, the informed consent for the placement of the said catheter was delivered in case they were assigned to the experimental group.

Those with a forecast hospitalization of less than one week, those who received palliative or symptomatic treatment, and those who were carriers of a CVC were not included in the study. Subsequently, at the time of placement of the midline catheter, those subjects who presented difficulty in venous access to insert the device after two consecutive attempts were excluded.

### 2.3. Interventions

In the case of patients in the control group, the peripheral catheter was the one used regularly in the hospitalization unit, Braun Introcan Safety^®^, an intravenous cannula with a genuinely passive safety device that is activated automatically and cannot be bypassed.

In the experimental group, the Leader Cath 20G^®^ midline catheter was used. It comprises three parts: a stainless-steel needle to perform the puncture, a straight metal guide used as a guarantor, and an 11 cm polyurethane catheter with a flexible flap in its upper back to facilitate its fixation.

The placement and maintenance of the PIVC were carried out following the CPG and the protocols established in the hospital. In the case of midline catheters, an insertion and maintenance protocol was drawn up following the existing CPGs at the time of study design.

### 2.4. Outcomes

The results to be evaluated would be the incidence of adverse events related to the catheter, especially phlebitis, the reason for withdrawing the catheter, the duration of cannulation, and the number of punctures performed.

### 2.5. Sample Size

The formula based on proportions comparison between the control and experimental groups was used to calculate the sample size. Data on the incidence of phlebitis in our unit (9.76%) were used. An expected proportion/incidence was established in the control group based on the data obtained in the literature review (3%).

For the sample size calculation, the statistical program Epidat 4.0 has been used, which indicates that the said size should be 408 subjects (204 subjects in each group) for a study with a power of 80%. It was planned to develop a pilot study to test the materials initially and from which modifications to the design would start, with 20 subjects in each group.

### 2.6. Randomisation

When the patient was admitted to our unit, a number was assigned from the admission department based on their admission order; that number was used for subsequent random assignment to the control or the experimental group.

The numbers corresponding to the subjects that will be part of each group were obtained through a website that generates random numbers.

The list was reviewed daily in the morning shift, and patients who did not give their informed consent and did not meet some of the other inclusion criteria in the experimental group were eliminated.

The assignment to one group or another was made by one of the researchers, who did not participate in the placement of the catheter or the subsequent data collection.

The list of random numbers was delivered daily to the staff performing the placement of the midline catheter (two nurses from the unit trained in the placement and handling of this type of device).

### 2.7. Blinding

Being an unblinded trial, only M.V.N., the researcher who performed the statistical analysis, was blinded (working with anonymized data). This researcher was different from the one who collected the data and could easily identify the device used because they were different.

Neither care providers nor patients could be blinded because it is easy to identify the device.

### 2.8. Statistical Method

Univariate descriptive analysis (using measures of central tendency or frequency distribution) and bivariate ones (using non-parametric tests) were performed. The Spearman coefficient was used to analyze correlations among numerical variables. The non-parametric Mann-Whitney or Kruskal-Wallis U tests were used to compare means among groups. In the analysis of relationships among categorical variables, the chi-square was used. In all calculations, a confidence interval of 95% has been considered. The effect size was calculated using Cohen’s d.

Data analysis was performed with the statistical analysis software IBM^®^ Statistical Package for Social Sciences (SPSS) v.22 (https://www.ibm.com/support/pages/spss-statistics-220-available-download (accessed on 26 October 2022).

## 3. Results

### 3.1. Participants’ Flow

**Figure 1:**
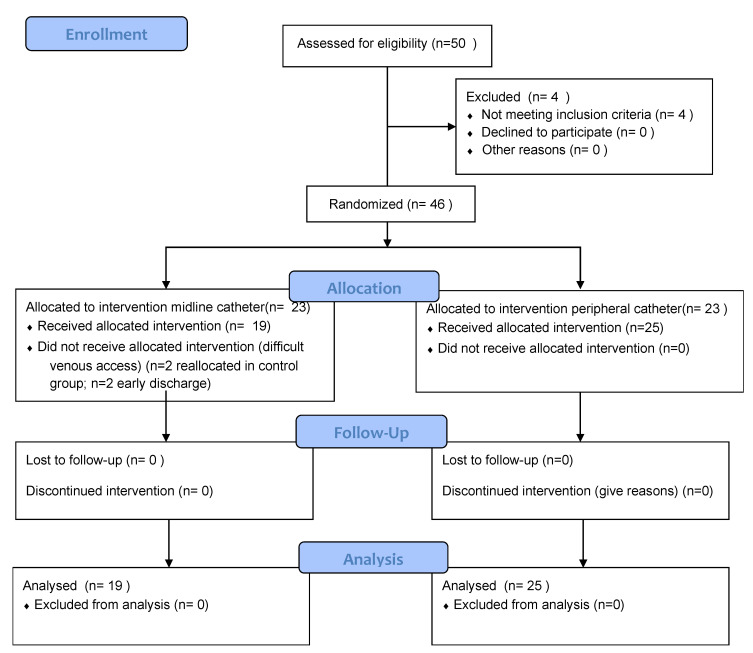
Participant flowchart.

Figure 1 shows participants’ flow according to consort guidelines.

As for a general description of the population, the average age was 76.45 years, the Charlson Comorbidity Index (CCI) was 4.11, and the subjects were primarily men, that is, there were 25 male subjects, representing 56.8%. Table 1 shows the characteristics of the participants according to the group to which they were assigned.

No statistically significant differences were identified in terms of the characterizing variables of the participants between both groups, which is indicative of their homogeneous composition.

### 3.2. Outcomes

#### 3.2.1. Catheters’ Related Features

The different variables related to the used catheters are shown in Table 2. The number of canalizations and phlebitis was inferior in the experimental group.

#### 3.2.2. Relationships between the Type of Catheter and Associated Complications

When studying the differences between the two groups using the Mann-Whitney U test, we found statistically significant differences both in the mean number of canalizations (more minor in the case of midlines) and in the days of duration of the canalization, both with a *p*-value < 0.01.

Correlations in both groups between numerical/ordinal variables were analyzed, finding in the case of the control group (PIVC) a statistically significant direct relationship between the time a catheter remained channeled and the level of phlebitis (rho = 0.612; *p* < 0.01). Regarding the experimental group, it was found a relationship between the variable duration of admission and the variables duration of cannulation (rho = 0.457; *p* < 0.05) and level of phlebitis (rho = 0.582; *p* < 0.01).

No serious adverse events related to the use of catheters were identified in either group of participants.

## 4. Discussion

At the beginning of the study, it was set out to assess the presence of complications related to intravenous therapy and the duration of days of midlines and PIVC in an inpatient unit. An experimental, randomized, longitudinal pilot study was designed to achieve this objective.

As a first approximation of this research, the midlines have presented a lower number of complications with a longer duration than the PVCs, far from their estimated life (although they were withdrawn at the discharge of the patients and not due to complications) and consistently superior to that of the conventional catheters.

Regarding the studied population studied, it has been verified that the PIVC have a useful life (time until their removal) of 3.22 days. This duration is estimated to be the recommended time for these catheters to stay. International organizations such as the Intravenous Nurses Society (INS) and the Centers for Disease Control (CDC) recommend a maximum length of stay for PIVC between 72 and 96 h to reduce the presence of complications [16]. On the other hand, and for this study, the midlines have remained inserted for an average of 8.22 days (in most cases, the time that the patient’s hospitalization and intravenous therapy have lasted). The INS and the CDC recommend this type of catheter for intravenous therapy longer than 6 days [16], so they would be indicated in this population. This duration time is also similar to that estimated by other studies that determine duration intervals from 7.69 to 16.4 days [4,5,34]. However, the actual useful life of these devices in this study could not be specified since the main reason for their withdrawal was hospital discharge. However, it must be taken into account that some articles indicate that these midlines can remain inserted for up to 20 days [22,35], 4 weeks [36], 29 days [14], or even 49 days [15].

Some complications and adverse events of the use of catheters include problems such as phlebitis, obstruction, infiltration, and extravasation [27,37]. Several risk factors have been found in different studies: high income, the size of the catheter, the use of medication, and the increase in the time of permanence, among others [27]. In this research, it has been found a relationship between the time of catheter insertion and the level of phlebitis. In addition, in the case of the PIVC, the correlation between both variables is statistically significant.

According to the results, the most frequent complications of PIVC are phlebitis and extravasation, both with 18.4%. In contrast, midlines present phlebitis as the most frequent complication, with 17.4%. However, their main reason for withdrawal was hospital discharge (30.4%), as well as studies carried out in our countries, such as Fortes Escalona, in which the leading cause of withdrawal is the discharge of the patient or the end of treatment [18].

Different studies have related the frequency of infection associated with the use of catheters with the number of insertion attempts since each puncture increases the risk [38,39]. For this reason, it is important to use devices that reduce the number of insertions. In this case, it can be observed how the number of cannulations is lower in the experimental group (3.88 ± 2.99 in CIVP vs. 1.47 ± 0.51 in midlines). In many cases, the patient receives single cannulation throughout the whole period of hospitalization. The longer a device remains in place, the fewer punctures will be necessary. People will suffer fewer venipunctures and, therefore, less pain, improving their quality of life and perception of intravenous therapy [40]. Indicators show that intravenous catheters in the medium term can favor the quality of care and that the procedures for inserting these vascular access devices are well tolerated [41].

It should be borne in mind that different studies that analyze the use of midlines justify their effectiveness not only by the adequate selection of the type of catheter but also by their early insertion [34]. For this reason, in this RCT, it was proposed that the midline insertion would not be extended beyond 48 h after hospitalization, coinciding with the change of PIVC established in the emergency department [28,35].

Even so, the importance of training nursing professionals to achieve vascular access must not be forgotten. Training has been improving in recent years in health centers with the creation of vascular access units and the training of professionals. Nursing practice in ultrasound-guided intravenous catheter cannulation, which, when applied to the insertion of PIVC, has shown a reduction in the insertion of central access devices, whose complications are greater [36,40,42].

## 5. Limitations

The principal limitation of this study is the small sample size of 44 subjects (and only 126 catheters). However, despite this the study having been designed as an RCT, the results obtained can be used as indications in future more extensive research, since most of the studies consulted establish comparisons between CVC and LPC or midlines but not between the types of peripheral venous devices.

This work shows the results of using a new device in a healthcare environment. However, since it is a pilot study, inferences cannot be made beyond this population and in this healthcare environment.

The registration of the RCT in clinicaltrials.gov was carried out after this pilot study, which could have made it challenging to put possible modifications in the protocol into practice before this pilot study.

## 6. Conclusions

Midline catheters have shown a longer duration of cannulation and fewer complications than conventional catheters. A longer duration causes a smaller number of punctures; therefore, in people with lengthy hospitalization and easy venous access, midline catheters could be used to reduce the number of punctures and the pain they cause.

## Figures and Tables

**Table 1:** Description of the characteristics of the population according to the assigned group.

		Control Group (CG)	Experimental Group (EG)	*p*-Value
*n*		25 (56.8%)	19 (43.2%)	
Age		75.80 ± 13.62 ¥	77.32 ± 14.94 ¥	0.60 ×
CCI		4.12 ± 2.20 ¥	4.11 ± 2.13 ¥	0.95 ×
Sex	Male	11 (44%) §	14 (75.7%) §	0.05 ××
	Female	14 (56%) §	5 (24.3%) §	
Days of admission		12.80± 11.36 ¥	19.89± 14.31 ¥	0.08

¥ Mean ± Standard deviation; § Frequency (Percentage); × Mann-Whitney test; ×× Chi-squared test.

**Table 2:** Catheter’s related features in the control group and experimental group.

		Control Group (CG)	Experimental Group (EG)	*p*-Value	Effect Size
Number of catheters		103 (81.7%) ^§^	23 (18.3%) ^§^	<0.01	
Number of canalizations		3.88 ± 2.99 ^¥^	1.47 ± 0.51 ^¥^	<0.001	0.660
Canalization duration		3.22 ± 2.28 ^¥^	8.13 ± 5.36 ^¥^	<0.001	0.571
Reason for withdrawal	Phlebitis	19 (18.4%) ^§^	4 (17.4%) ^§^		
	Obstruction	4 (17.4%) ^§^	3 (13%) ^§^		
	extravasation	11 (10.7%) ^§^	3 (13%) ^§^		
	Accidental removal	3 (13%) ^§^	3 (13%) ^§^		
	Malfunction	19 (18.4%) ^§^	2 (8.7%) ^§^	<0.05	
	Discharge	3 (13%) ^§^	7 (30.4%) ^§^		
	Other	2 (1.9%) ^§^	1 (4.3%) ^§^		
Phlebitis level	Level 0	84 (81.6%) ^§^	18 (78.3%) ^§^		
	Level 1	4 (3.9%) ^§^	1 (4.3%) ^§^	0.95	0.192
	Level 2	14 (13.6%) ^§^	4 (17.4%) ^§^		
	Level 3	1 (0.9%) ^§^	0 (0%) ^§^		

^¥^ Mean ± Standard deviation; ^§^ Frequency (Percentage).

## Data Availability

The data presented in this study are available on request from the corresponding author.
